# The relationship between socio-demographic factors and depression: a cross sectional study among civilian participants in hostilities in Ukraine

**DOI:** 10.1038/s41598-023-49289-6

**Published:** 2023-12-11

**Authors:** Stanisław Fel, Krzysztof Jurek, Katarzyna Lenart-Kłoś

**Affiliations:** The John Paul II Catholic University of Lublin, Lublin, Poland

**Keywords:** Human behaviour, Quality of life

## Abstract

There is still a gap in scientific knowledge in relation to civilian participants in hostilities. This is despite the fact that there is an extensive body of literature on major depressive disorder (MDD) in individuals who have experienced armed conflict. The purpose of this article is to identify socio-demographic factors which are related to levels of depression among civilian participants in the war in Ukraine, based on a cross-sectional study that was conducted in 2019 from a convenience sample of 314 Ukrainian adults (235 males). Depression was assessed via the Beck Depression Inventory. Multiple regression analyses were conducted to identify possible predictors of depression. Significant predictors were: loss of a loved one, place of residence, age, health insurance, financial situation, and marital status (*F* (6, 224) = 10.515, *p* < 0.001, *R*^2^ = 0.21; *Adjusted R*^2^ = 0.19). They also show that symptoms of depression resulting from the loss of a loved one due to war can be reduced through participation in an educational system. Having children is associated with a risk of more severe depression. Specialists are encouraged to engage in face-to-face interviews and to maintain a supportive and safe environment for participants in hostilities, e.g., in the area of education.

## Introduction

Between 1989 and 2015, about 1.45 billion people experienced war, and approx. 1 billion of those were adults. Among adult war survivors living in post-conflict areas 242 million were affected by post traumatic stress disorder (PTSD), while major depressive disorder (MDD) affects an estimated 238 million adult war survivors^1^. Another systematic review reported that in 2019 there were about 316 million adult war survivors suffering from MDD and/or PTSD^2^. MDD is one of the most common mental disorders in war-ridden communities, especially when compared to communities which have never experienced armed conflict^3^. War-related depression has been indicated as resulting from, among other things, long-term exposure to severe stress^4,5^. Studies have indicated that MDD and PTSD tend to co-occur^6,7^. PTSD and MDD often appear in response to traumatic experiences^8^.

A study conducted among internally displaced people (IDPs) in Ukraine and people who were not affected by the war indicates that the prevalence of depression was higher (25%) among IDPs than the general population (14%)^9^. A study performed in 2022 showed that 88.3% of Ukrainians were distressed by war noises; 36% reported poor sleep quality, 50.1% felt angry with the war, and 59.2% developed a mental health problem due to the war^10^. According to an online survey carried out in Ukraine during the initial period of the Russian invasion (March 19–31, 2022), 46.8% of Ukrainian adults reported symptoms of depression^11^. Another study among Ukrainian adults aged 18–65 showed that 42.3% of participants suffered from symptoms of depression. Among them, prevalence of severe depressive symptoms was 16.4%^12^. It can be considered potential barriers to posttraumatic growth^13^. Adolescents from the war-torn Donetsk region reported a higher level of depression than adolescents from Kirovograd in central Ukraine (a relatively peaceful region; according to a study conducted in 2016–2017). Mild/moderate depression was experienced by 28.2% of adolescents from the Donetsk region and 25.2% of adolescents from central Ukraine, and moderate severe depression was experienced respectively by 7.5% and 2.9%^14^ of adolescents.

The issue that makes individuals susceptible to developing MDD is not only their exposure to conflict or war itself (e.g. lost loved one), but also additional factors that can aggravate trauma-related symptoms (e.g. one's place of residence)^15^. For example, many Ukrainians had to leave their previous place of residence, which also resulted in the need to look for a new place of employment. Many times, the surveyed Ukrainians, in addition to continuing their education at a given level (e.g., high school), had to retrain or complete vocational courses. All these situations are understood as a “continuation of education”. The education does not only refer to school-age students. Nowadays, much emphasis is placed on the idea of lifelong learning and readiness to adapt to the labour market and changes found in different stages of professional life. The war demonstrated this vividly. People who were displaced by the impacts of war left their belongings, businesses and often started over, with the first step being to learn a new profession and acquire new qualifications, often through extracurricular education. In addition to any war-related trauma, what can contribute to the development and aggravation of MDD includes poor socio-economic conditions (financial security, savings) and other factors unrelated directly to war (having children or marital status)^16^.

The situation of war-affected civilians in Ukraine remains an unexplored field of research. The actions carried out by the Russian side until 2022 were mostly described in terms of hybrid warfare, new generation warfare, proxy war, or information warfare^17^. This method of warfare introduces non-military measures, which include economic sanctions, airspace, naval, and/or land blockades, political isolation, and threats of use of force, to implement what is known as an indirect action strategy.The situation changed dramatically on February 24, 2022, when Russia launched a full-scale invasion of Ukraine, using conventional weapons. The civilian population of Ukraine has been the target of attacks and war crimes^18^. Russian attacks launched on February 24, 2022, have resulted in the mass migration of civilians, mostly women and children, to Poland^19,20^. This new context calls for further analysis and research on depression among civilian participants in the war in Ukraine,.

## Methods

### Study design

A cross-sectional survey using a convenience sampling method was used to collect data. Answers were obtained from adult Ukrainian civilians in Donbass and displaced people from Donbass living in the central or western parts of Ukraine^21^. To ensure safety for both respondents and survey implementers, due to research being also carried out in war zones, respondents were recruited the non-probability snowball. Research was conducted in 2019 in hospitals, readaptation centres, and other places where the displaced persons resided. The researchers supervised the data collection process.

### Procedure

Respondents were briefed on the purpose of the research. Participation in the study was anonymous and voluntary. The study was conducted by trained interviewers of Ukrainian nationality. The study was supervised by the researchers of the Institute of Sociological Sciences of the John Paul II Catholic University of Lublin. Participants responded to a questionnaire package during face-to-face interviews carried out in Ukrainian language. Collected data was reviewed and checked for completeness before data entry, while incomplete questionnaires were discarded.

### Data measurement

Each respondent was asked to complete the Polish version of the Beck Depression Inventory (BDI)^22^. This consists of 21 points evaluated on a scale from 0 to 3. The tool can be used to assess the strength of individual depressive symptoms, such as bad mood, anxiety, fear, low self-esteem, loss of the ability to experience happiness, feelings of guilt, an expectation of punishment, self-dislike, suicidal thoughts, tearfulness, feelings of anxiety, and the loss of libido. The BDI demonstrates high internal consistency, with alpha coefficients of 0.86 and 0.81 for psychiatric and non-psychiatric populations, respectively. The Cronbach’s alpha coefficient of this study was 0.83.

### Statistical analysis

Statistical analysis was carried out with IBM SPSS Statistics, version 25. In our statistical analysis, we used parametric tests because of the normal and homogeneous distribution of the data (assumptions of normality were checked using the Shapiro–Wilk test, homogeneity of variance was assessed using Levene’s test). Skewness was within ± 1 and kurtosis was within ± 2. Two sample t-tests were conducted to compare BDI scores for independent variables with two levels. One-way analyses of variance (ANOVA) were conducted to compare the BDI score of more than two groups. Effect sizes were reported. Two-way ANOVA was used to determine the effect of two nominal predictor variables on BDI score. Residues showed normal distribution. The equality of variances was confirmed (p > 0.05). Multiple linear regression analysis (the stepwise method) was used to identify variables that predict the BDI score (introducing into the model the variables shown in Table 2). To assess multicollinearity, the variance inflation factor (VIF) was calculated. The value of all variables was in the acceptable range (1.045–1.124). In addition, assumptions of linearity and homogeneity of variance were checked using scatter plots. The level of significance was considered as *p* < 0.05.

### Ethical approval

The study was conducted in accordance with the Declaration of Helsinki and approved by the Research Ethics Committee of the Institute of Sociological Sciences of the John Paul II Catholic University of Lublin (protocol code: KEB-IS-3/2019).

### Informed consent

Informed consent was obtained from all subjects involved in the study. The study did not involve participants under the age of 18.

## Results

The study involved 314 participants. The mean age was 34.08 (SD = 9.83, range 18–74). Moderate or severe depression was found in 12.8% of the sample (Table 1).

**Table 1:** Depression levels among participants in the war in Ukraine.

Depression level	N	%
No depression	173	55.1
Mild depression	101	32.2
Moderate depression	20	6.4
Severe depression	20	6.4
Total	314	100.0

Statistically significant differences were found for depression levels in relation to age, having children, continuing education, place of residence, financial situation, loss of a loved one, health insurance, and having savings (Table 2). No significant differences were found in BDI scores (p > 0.05) in relation to gender, marital status, education level, distance from the war zone, or financial security.

**Table 2:** Parametric test results for BDI score.

Variables		n	M	SD	Value^a^	Effect size
Whole group		314	11.00	8.45		
Age	≤ 25	62	8.39	8.40	2.891**	0.06
	26–30	63	10.95	8.50		
	31–35	62	10.37	7.38		
	36–40	43	12.49	7.67		
	41–45	29	14.67	10.78		
	46–50	27	10.93	6.71		
	> 50	17	14.83	9.20		
Gender	Male	74	10.60	8.05	− 1.642	–
	Female	232	12.45	9.58		
Marital status	Married/cohabitating	127	11.14	7.64	− 0.306	–
	Single/separated/divorced/widow(er)	172	11.45	9.55		
Children	No	192	9.08	8.20	− 3.072**	0.36
	Yes	116	12.09	8.45		
Education	Primary/secondary education	165	11.47	8.90	1.277	–
	Higher education	132	10.21	7.94		
Continuing education	No	68	11.80	8.38	3.198**	0.44
	Yes	240	8.13	8.29		
Place of residence	Rural	67	14.73	10.68	4.142***	0.57
	Urban	236	9.98	7.48		
Financial situation	Bad	144	12.90	9.49	3.825***	0.45
	Good	163	9.23	6.97		
Lost loved one	No	57	10.20	8.12	− 3.695***	0.54
	Yes	245	14.74	9.30		
Distance from hostilities	≤ 500 km	71	9.37	1.11	0.014	–
	501–999 km	171	8.43	0.64		
	≥ 1000 km	41	8.40	1.30		
Health insurance	No	54	11.75	8.30	3.414***	0.51
	Yes	255	7.50	8.41		
Savings	No	75	11.83	8.48	2.982**	0.40
	Yes	233	8.53	7.87		
Financial security	No	145	11.19	8.78	0.467	–
	Yes	163	10.74	8.09		

^a^Two sample t-tests or one-way analyses of variance (ANOVA); *p < 0.05; ** *p* < 0.01; ***p < 0.001.

**Figure 1:**
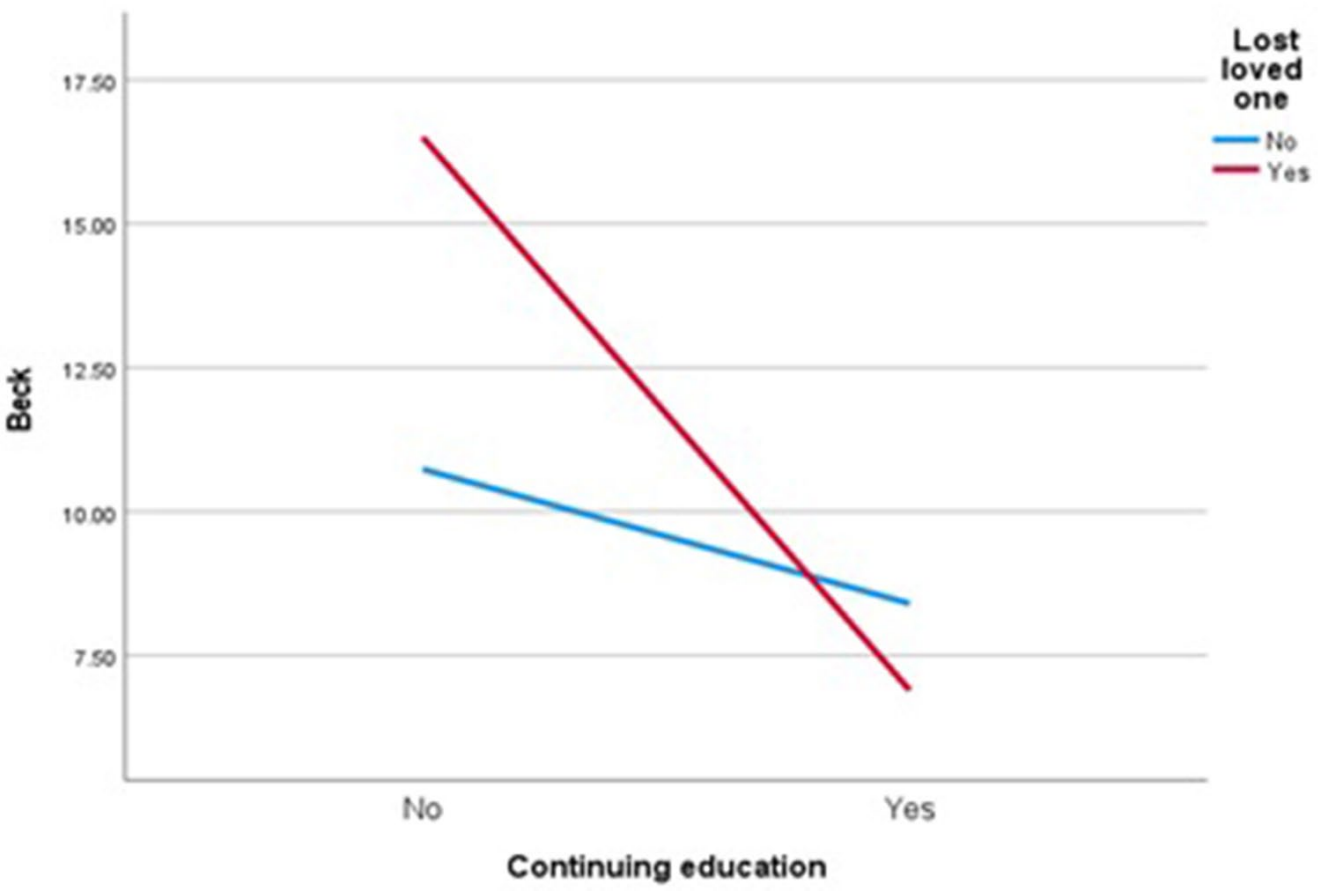
The effect of loss of loved one and continuing education on BDI score.

The primary effect of continuing education was statistically significant (*F* = 14.589; *p* < 0.001; *η*^2^ = 0.05, moderate effect). The effect of the loss of a loved one was not statistically significant (*F* = 1.859; *p* = 0.174). Instead, the opposite was true for the effect of interaction (*F* = 5.410; *p* = 0.021; *η*^2^ = 0.02, small effect). Participants in war who had lost a loved one and continued education had lower levels of depression than those who had lost a loved one but did not continue education (Fig. 1).

**Figure 2:**
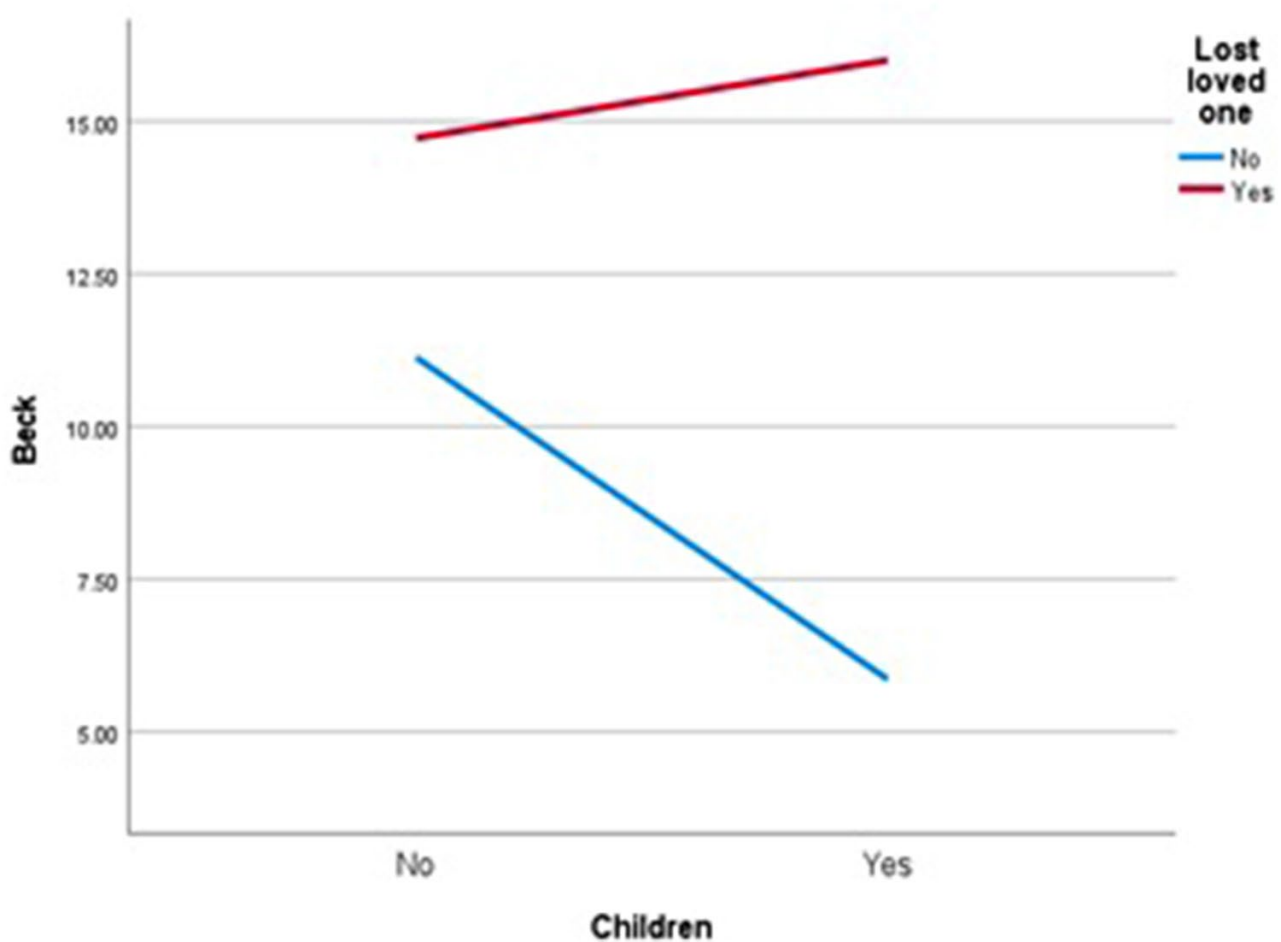
The effect of loss of a loved one and of having children on BDI score.

In the second model, the variables were the loss of a loved one (Yes vs. No) and having children (Yes vs. No). The primary effect was established for the loss of a loved one (*F* = 5.661; *p* = 0.018; *η*^2^ = 0.02, a small effect). The primary effect of having children was also statistically significant (*F* = 10.512; *p* < 0.001; *η*^2^ = 0.04, a small/moderate effect). The effect of interaction was statistically significant (*F* = 4.544; *p* = 0.034; *η*^2^ = 0.02, a small effect). Having children increased levels of depression among individuals who had lost a loved one. In people who had not lost a loved one but had children, depression levels decreased (Fig. 2).

The regression model (*F* = 10.515, *p* < 0.001) predicted 26% of the sample outcome variance with a coefficient of determination (Adj. *R*^2^ = 0.19). The final model includes six significant predictors of depression—loss of a loved one (*β* = 0.127, *t* = 2.173, *p* < 0.001), place of residence (*β* = − 0.249, *t* = − 4.308, *p* < 0.001), age (*β* = 0.206, *t* = 3.110, *p* = 0.002), marital status (*β* = 0.162, *t* = 2.485, *p* = 0.014), financial situation (*β* = − 0.149, *t* = − 2.526, *p* = 0.012), and health insurance (*β* = − 0.159, *t* = − 2.670, *p* = 0.008). The model predicted lower levels of depression for participants in war who lived in urban areas, were in relationships, and had health insurance. Higher levels of depression were associated with the loss of a loved one, older age, and a poorer financial situation (Table 3).

**Table 3:** Multiple regression analysis predicting BDI score (final model).

	Unstandardised coefficients		*Beta*	*t*	95% CI		Collinearity	
	B	S.E			Lower	Upper	Tolerance	VIF
Constant	9.795	2.638	–	3.713***	4.598	14.992	–	–
Place of residence	− 5.453	1.266	− 0.249	− 4.308***	− 7.946	− 2.960	0.981	1.019
Health insurance	− 3.619	1.355	− 0.159	− 2.670**	− 6.289	− 0.949	0.927	1.079
Lost loved one	2.896	1.333	0.127	2.173*	0.271	5.521	0.959	1.043
Financial situation	− 2.620	1.037	− 0.149	− 2.526*	− 4.662	− 0.577	0.941	1.062
Age	0.181	0.058	0.206	3.110**	0.066	0.296	0.748	1.337
Marital status	2.885	1.161	0.162	2.485*	0.598	5.171	0.775	1.290

R = 0.455; *R*^2^ = 0.207; *Adjusted R*^2^ = 0.188.

*p < 0.05; **p < 0.01; ***p < 0.001.

## Discussion

The aim of this article has been to identify socio-demographic factors which are related to lower levels of depression among the civilian participants in the war in Ukraine.

Our findings support and corroborate the results of previous studies that experiencing multiple traumatic events, including acts of violence, death threats, missing or loosing loved ones, witnessing death, and post-migration stressors^23^ increases the risk of mental health issues. It is difficult to estimate the proportion of refugees at risk of chronic or pathological grief who might experience clinically significant difficulty in coping with the death of their loved ones. Moreover, the risk factors and susceptibility related to pathological grief remain relatively unknown in literature on grieving refugee populations. Beyond all doubt, as exemplified by the war in Ukraine, civilians are the most prevalent of war casualties^24^. What determines depression is not only the loss of loved ones but also their disappearance during war. Another research demonstrated significantly higher levels of depression, anxiety, and somatisation in women who had some of their family members gone missing when compared to women who had no such experiences. Higher levels of depression, anxiety, and somatisation were found in women who had missing children when compared to other missing family members^25^. Our study shows that the levels of depression in individuals who lost a loved one but were able to continue their education were lower than in those who were not able to.

Many studies, including those conducted by international organisations such as the WHO, prove that actions supporting children and young people’s mental health are usually carried out in schools (e.g. in low- or medium-income countries affected by war and violence, where there is a high risk of mental health problems). In addition, experts have argued that schools/universities provide favourable opportunities for interventions that should cover entire populations/entire communities. Our study, as conducted among adults, confirms this theory. Moreover, some studies show that, for child-soldiers, the continuum of risk factors and factors protecting mental health include educational opportunities^26^. We indicate that participants in war, educational opportunities are a kind of protective resource. It can be assumed that they bring benefits in many dimensions. Schools and, more broadly, the school environment play an integral role in supporting the development of social, emotional, and cognitive skills, the effects of which can contribute to short-term and long-term mental health. Moreover, our study draws attention to the relationship between the loss of a loved one and having children in the context of depression level. The participants in warfare who had children and had lost a loved person experienced higher stress than childless individuals. This result suggests that—in the context of war—having children not only does not act as a protective factor but actually heightens mental strain. In most cases, the loss of a loved one is a profound experience, causing an emotional crisis that triggers various psychological processes and phenomena. Death as a result of war intensifies anxiety about the health and life of other family members, including children.

We also show that financial (socio-economic) status and ability to access healthcare (having health insurance) determine depression level. Whatever their previous socio-economic status, war refugees often leave behind all their property, including businesses and real estate they rely on for their lives, savings, and even documents which confirm their professional qualifications. As a result, many of them arrive in host countries in relative poverty and can stay poor for many years. To a large degree, this depends on the host community. In the context of the war in Ukraine, Poland showed an open attitude by accepting refugees without any limitations, and providing them with social and financial support (housing, food, clothing, employment). A meta-analysis of 59 studies which compared the mental health of refugees with that of residents showed a clear linear correlation between the mental health of refugees and their financial performance, influenced by the right to work, access to employment, and socio-economic status^27^. For example, depressive symptoms among Ukrainian refugees living in Germany are associated with a lower quality of life of the refugees in the country^28^. On the other hand, research conducted among Syrian refugees showed that post-migration conditions and positive perspectives in host countries may prevent mental health disorders^29^. A lower financial status can also be associated with one's professional situation. A study conducted among persons who were internally displaced after post-election violence, living in a Hajj camp in Kaduna, northern Nigeria, showed that unemployed or retired individuals, i.e. those with lower or no income, had a higher rate of definitive depression^30^. The ability to access healthcare plays an important role for the mental health of war-affected people. A study carried out among displaced Afghans shows that both able-bodied and disabled individuals experience depression (67.7% vs. 71.7%)^31^. The possibility of receiving healthcare also depends on legal conditions. For example, Syrian refugees struggled with restricted rights in Lebanon, which has reduced their access to adequate healthcare and education^32^. In contrast, receiving financial support and having access to social and healthcare services has contributed to better mental health outcomes for Syrian refugees with residence permission in Germany^33^. Our study confirms the current state of knowledge, according to which worse financial situation is associated with a higher level of depression. Additionally, we have found that one of the important factors is health insurance. Access to specialist treatment or medical services may involve high costs, having insurance is a significant protective resource in a situation of war. Being insured reduces stress and anxiety concerning access to health care. In the case of health insurance, the patient can expect reimbursement for treatment and medication expenses. It should be remembered that, so far, the Ukrainian system is formally functioning in accordance with the organizational assumptions of the Semashko model, with central budgetary financing, a lack of pro-effective solutions in the financing of medical services (global budgeting of health units), hierarchical organizational structure, and a dominance of the public sector. It should be remembered that Ukrainian system is so far formally functioning in accordance with the organizational assumptions of the Semashko model, with central budgetary financing, lack of pro-effective solutions in the financing of medical services (global budgeting of health units), hierarchical organizational structure, and dominance of public sector^34^.

Our study shows that the level of depression is related to age, place of residence and marital status. The majority of studies, of which ours can also be included, report that the higher the mean age of war-affected populations, the higher their depression levels. A research conducted in all former Yugoslavian countries showed that the higher a person’s age (from above 40 years old) the greater their risk of depression^35^. A study on Syrian refugees displaced to Sweden also showed that depression was more prevalent among older people^36^. Similar findings were reported for Syrian refugees residing in the Kurdistan region of Iraq^37^ as well as for Syrian refugees in Lebanon (who were over 45 years of age)^29^. Our findings demonstrate that living in a rural area is correlated with higher levels of depression. However, there is no agreement in the literature regarding whether the place of residence has an impact on depression. A study carried out among young people in Kosovo, formerly a war theatre, showed that girls living in rural areas are more likely to experience stress and require more attention from professionals. What is more, a study conducted among Syrian refugees in Iraq showed that growing up in urban areas contributes to PTSD, but it makes no difference on depression levels. Nonetheless, one should consider that fights in Syria took place mainly in big cities, which makes it difficult to compare urban and rural communities^37^. Another study, conducted among veterans with at least 1 warzone deployment in central and north-eastern Pennsylvania showed that female veterans living in non-rural areas had an increased risk of depression in comparison to those in rural areas^38^. Yet, what is important to consider is that, although it is changing with growing digitisation, veterans from rural areas often have poorer access to medical care^39^. Finally, being married also reduces the risk of experiencing depression. A study conducted amongst internally displaced persons in northern Uganda^40^ shows that married individuals are less likely to develop depression when compared to the divorced, separated, or widowed/widowered. Additionally, a study on war veterans and their wives shows that the satisfaction of wives from their marriage with war veterans depends on the wife’s depression levels^41^. Among United States' male Veterans from Operations Enduring/Iraqi Freedom (OEF/OIF), a third reported having divorced or separated as a result of their involvement in war operations. Compared to veterans who were able to stay married, those divorced or separated as a result of war operations experienced intensified stress during and after deployment as well as significantly less social support^42^. A study conducted among female veterans of the United States military also shows that relationship status is correlated with diagnosed depression. Female veterans who were not in a relationship (i.e., divorced, widowed, or single) were 1.3 times more likely to be diagnosed than those in partnerships (i.e., married, cohabiting, or seriously-dating)^43^. Lastly, according to a study conducted among Syrian refugees in Lebanon, being divorced or separated was more likely to result in more significant symptoms of depression^29^. There is no agreement among researchers regarding the significance of demographic factors such as age or place of residence. Our study supports the existing knowledge about depression among participants in war, according to which depression level increases with age and is higher among the inhabitants of villages and single individuals.

## Limitations

There are several limitations that should be taken into account when interpreting the results of our study. The cross-sectional nature of the study did not allow the establishment of any temporal relationships. Additionally, non-probability snowball sampling technique was applied to recruit the required participants. The participants were not randomly selected. These solutions undermine the possibility to make generalizations based on the sample. Another limitation of this study is related to the data collection method. The self-report surveys increase the possibility of having answers containing unaddressed bias. Individuals may feel pressured to give a more socially acceptable and less truthful answer, or might not have been able to make an accurate assessment of their situation.

## Conclusion

Our data reveal a significant predictive function of financial (socio-economic) status and socio-demographic factors for the BDI score. The study may help to healthcare providers (including social workers and caregivers) goals guiding therapeutic support. Specialists are encouraged to engage in face-to-face interviews and to maintain a supportive and safe environment for participants in hostilities, e.g., in the area of education.

## Data Availability

The data presented in this study is available on request from the corresponding author.

## References

[1] Charlson F, van Ommeren M, Flaxman A, Cornett J, Whiteford H, Saxena S (2019). TNew WHO prevalence estimates of mental disorders in conflict settings: A systematic review and meta-analysis. Lancet.

[2] Hoppen TH, Priebe S, Vetter I, Morina N (2021). Global burden of post-traumatic stress disorder and major depression in countries affected by war between 1989 and 2019: A systematic review and meta-analysis. BMJ Glob. Health.

[3] Steel Z (2009). Association of torture and other potentially traumatic events with mental health outcomes among populations exposed to mass conflict and displacement: A systematic review and meta-analysis. JAMA.

[4] Momartin S, Silove D, Manicavasagar V, Steel Z (2004). Comorbidity of PTSD and depression: Associations with trauma exposure, symptom severity and functional impairment in Bosnian refugees resettled in Australia. J. Affect. Disord..

[5] Yousef L (2021). War-related trauma and post-traumatic stress disorder prevalence among Syrian university students. Eur. J. Psychotraumatol..

[6] Morina N, Hoppen TH, Priebe S (2020). Out of sight, out of mind: Refugees are just the tip of the iceberg. An illustration using the cases of depression and posttraumatic stress disorder. Front. Psychiatry.

[7] Breslau N (1998). Trauma and posttraumatic stress disorder in the community: The 1996 Detroit Area Survey of Trauma. Arch. Gen. Psychiatry.

[8] Madianos MG, Sarhan AL, Koukia E (2011). Posttraumatic stress disorders comorbid with major depression in West Bank, Palestine: A general population cross sectional study. Eur. J. Psychiatry.

[9] Kuznetsova I, Mikheieva O, Catling J, Round J, Babenko S (2019). The Mental Health of Internally Displaced People and the General Population in Ukraine.

[10] Chudzicka-Czupała A (2023). Depression, anxiety and post-traumatic stress during the 2022 Russo-Ukrainian war, a comparison between populations in Poland, Ukraine, and Taiwan. Sci. Rep..

[11] Xu W (2023). Mental health symptoms and coping strategies among Ukrainians during the Russia-Ukraine war in March 2022. Int. J. Soc. Psychiatry.

[12] Kurapov A (2023). Six months into the war: A first-wave study of stress, anxiety, and depression among in Ukraine. Front. Psychiatry.

[13] LaRocca MA, Avery TJ (2020). Combat experiences link with posttraumatic growth among veterans across conflicts: The influence of PTSD and depression. J. Nerv. Ment. Dis..

[14] Osokina O (2023). Impact of the Russian invasion on mental health of adolescents in Ukraine. J. Am. Acad. Child Adolesc. Psychiatry.

[15] Henkelmann JR (2020). Anxiety, depression and post-traumatic stress disorder in refugees resettling in high-income countries: Systematic review and meta-analysis. BJPsych Open.

[16] Benjet C (2016). The epidemiology of traumatic event exposure worldwide: Results from the World Mental Health Survey Consortium. Psychol. Med..

[17] Fel S, Niewiadomska I, Lenart-Kłoś K (2022). People in the Face of Modern Warfare: Relationships Between Resource Distribution and Behaviour of Participants in the Hostilities in Ukraine.

[18] Baker MS, Baker J, Burkle FM (2023). Russia's hybrid warfare in Ukraine threatens both healthcare & health protections provided by international law. Ann. Glob. Health.

[19] Dzhus M, Golovach I (2022). Impact of Ukrainian–Russian war on health care and humanitarian crisis. Disaster Med. Public Health Prep..

[20] Kyliushyk I, Jastrzebowska A (2023). Aid attitudes in short- and long-term perspectives among Ukrainian migrants and Poles during the Russian war in 2022. Front. Sociol..

[21] Fel S, Jurek K, Lenart-Kłoś K (2022). Relationship between socio-demographic factors and posttraumatic stress disorder: A cross sectional study among civilian participants & hostilities in Ukraine. Int. J. Environ. Res. Public Health.

[22] Beck AT, Steer RA, Garbin MG (1988). Psychometric properties of the Beck Depression Inventory: Twenty-five years of evaluation. Clin. Psychol. Revi..

[23] Kokou-Kpolou CK (2020). Correlates of grief-related disorders and mental health outcomes among adult refugees exposed to trauma and bereavement: A systematic review and future research directions. J. Affect. Disord..

[24] Bartov O (2000). Mirrors of Destruction: War, Genocide, and Modern Identity.

[25] Baraković D, Avdibegović E, Sinanović O (2013). Depression, anxiety and somatization in women with war missing family members. Mater. Sociomed..

[26] Frounfelker RL (2019). Living through war: Mental health of children and youth in conflict-affected areas. Int. Rev. Red Cross.

[27] Hynie M (2018). The social determinants of refugee mental health in the post-migration context: A critical review. Can. J. Psychiatry.

[28] Buchcik J, Kovach V, Adedeji A (2023). Mental health outcomes and quality of life of Ukrainian refugees in Germany. Health Qual. Life Outcomes.

[29] Naal H (2021). Prevalence of depression symptoms and associated sociodemographic and clinical correlates among Syrian refugees in Lebanon. BMC Public Health.

[30] Sheikh TL (2015). Correlates of depression among internally displaced persons after post-election violence in Kaduna, North Western Nigeria. J. Affect. Disord..

[31] Cardozo BL (2004). Mental health, social functioning, and disability in postwar Afghanistan. JAMA.

[32] Blanchet K, Fouad FM, Pherali T (2016). Syrian refugees in Lebanon: The search for universal health coverage. Conflict Health.

[33] Georgiadou E, Zbidat A, Schmitt GM, Erim Y (2018). Prevalence of mental distress among Syrian refugees with residence permission in Germany: A registry-based study. Front. Psychiatry.

[34] Romaniuk P, Semigina T (2018). Ukrainian health care system and its chances for successful transition from Soviet legacies. Glob. Health.

[35] Priebe S (2010). Mental disorders following war in the Balkans: A study in 5 countries. Arch. Gen. Psychiatry.

[36] Tinghög P (2017). Prevalence of mental ill health, traumas and postmigration stress among refugees from Syria resettled in Sweden after 2011: A population-based survey. BMJ Open.

[37] Mahmood HN, Ibrahim H, Goessmann K, Ismail AA, Neuner F (2019). Post-traumatic stress disorder and depression among Syrian refugees residing in the Kurdistan region of Iraq. Conflict Health.

[38] Boscarino JJ (2020). Mental health status in veterans residing in rural versus non-rural areas: Results from the veterans’ health study. Mil. Med. Res..

[39] Boscarino JA, Larson S, Ladd I, Hill E, Paolucci SJ (2010). Mental health experiences and needs among primary care providers treating OEF/OIF veterans: Preliminary findings from the Geisinger Veterans Initiative. Int. J. Emerg. Ment. Health.

[40] Roberts B, Ocaka KF, Browne J, Oyok T, Sondorp E (2008). Factors associated with post-traumatic stress disorder and depression amongst internally displaced persons in northern Uganda. BMC Psychiatry.

[41] Klaric M (2011). Marital quality and relationship satisfaction in war veterans and their wives in Bosnia and Herzegovina. Eur. J. Psychotraumatol..

[42] Gros DF, Lancaster CL, Teves JB, Libet J, Acierno R (2019). Relations between post-deployment divorce/separation and deployment and post-deployment stressors, social support, and symptomatology in Veterans with combat-related PTSD symptoms. J. Mil. Veteran Fam. Health.

[43] Thomas KH (2016). Predictors of depression diagnoses and symptoms in United States female veterans: Results from a national survey and implications for programming. J. Mil. Veterans Health.

